# The Relationship Between a History of High-risk and Destructive Behaviors and COVID-19 Infection: Preliminary Study

**DOI:** 10.2196/40821

**Published:** 2023-04-14

**Authors:** Nicole L Vike, Sumra Bari, Khrystyna Stetsiv, Sean Woodward, Shamal Lalvani, Leandros Stefanopoulos, Byoung Woo Kim, Nicos Maglaveras, Aggelos K Katsaggelos, Hans C Breiter

**Affiliations:** 1 Department of Computer Science University of Cincinnati Cincinnati, OH United States; 2 Department of Psychiatry and Behavioral Sciences Northwestern University Chicago, IL United States; 3 Department of Electrical Engineering Northwestern University Evanston, IL United States; 4 Laboratory of Medical Informatics Aristotle University of Thessaloniki Thessaloniki Greece; 5 Department of Computer Science Northwestern University Evanston, IL United States; 6 Department of Radiology Northwestern University Evanston, IL United States; 7 Laboratory of Neuroimaging and Genetics Department of Psychiatry Massachusetts General Hospital and Harvard School of Medicine Boston, MA United States

**Keywords:** substance use, gambling, violent behaviors, COVID-19, destructive behaviors, mental health

## Abstract

**Background:**

The COVID-19 pandemic has heightened mental health concerns, but the temporal relationship between mental health conditions and SARS-CoV-2 infection has not yet been investigated. Specifically, psychological issues, violent behaviors, and substance use were reported more during the COVID-19 pandemic than before the pandemic. However, it is unknown whether a prepandemic history of these conditions increases an individual’s susceptibility to SARS-CoV-2.

**Objective:**

This study aimed to better understand the psychological risks underlying COVID-19, as it is important to investigate how destructive and risky behaviors may increase a person’s susceptibility to COVID-19.

**Methods:**

In this study, we analyzed data from a survey of 366 adults across the United States (aged 18 to 70 years); this survey was administered between February and March of 2021. The participants were asked to complete the Global Appraisal of Individual Needs–Short Screener (GAIN-SS) questionnaire, which indicates an individual’s history of high-risk and destructive behaviors and likelihood of meeting diagnostic criteria. The GAIN-SS includes 7 questions related to externalizing behaviors, 8 related to substance use, and 5 related to crime and violence; responses were given on a temporal scale. The participants were also asked whether they ever tested positive for COVID-19 and whether they ever received a clinical diagnosis of COVID-19. GAIN-SS responses were compared between those who reported and those who did not report COVID-19 to determine if those who reported COVID-19 also reported GAIN-SS behaviors (Wilcoxon rank sum test, α=.05). In total, 3 hypotheses surrounding the temporal relationships between the recency of GAIN-SS behaviors and COVID-19 infection were tested using proportion tests (α=.05). GAIN-SS behaviors that significantly differed (proportion tests, α=.05) between COVID-19 responses were included as independent variables in multivariable logistic regression models with iterative downsampling. This was performed to assess how well a history of GAIN-SS behaviors statistically discriminated between those who reported and those who did not report COVID-19.

**Results:**

Those who reported COVID-19 more frequently indicated past GAIN-SS behaviors (*Q*<0.05). Furthermore, the proportion of those who reported COVID-19 was higher (*Q*<0.05) among those who reported a history of GAIN-SS behaviors; specifically, gambling and selling drugs were common across the 3 proportion tests. Multivariable logistic regression revealed that GAIN-SS behaviors, particularly gambling, selling drugs, and attention problems, accurately modeled self-reported COVID-19, with model accuracies ranging from 77.42% to 99.55%. That is, those who exhibited destructive and high-risk behaviors before and during the pandemic could be discriminated from those who did not exhibit these behaviors when modeling self-reported COVID-19.

**Conclusions:**

This preliminary study provides insights into how a history of destructive and risky behaviors influences infection susceptibility, offering possible explanations for why some persons may be more susceptible to COVID-19, potentially in relation to reduced adherence to prevention guidelines or not seeking vaccination.

## Introduction

### Background

The SARS-CoV-2 pandemic has led to a concern about behavioral alterations in both those with COVID-19 and those dealing with pandemic-related stresses [1]. SARS-CoV-2 infection has been shown to cause COVID-19 morbidity and mortality with symptoms ranging from severe respiratory distress to prolonged cognitive dysfunction (eg, brain fog) and mental health (MH) problems [2-4]. In the United States, rates of MH conditions, drug overdoses, and violence-related emergency department visits were higher during the pandemic than during the previous year (2019) [5]. Reports also suggest an increase in high-risk behaviors, such as problematic web-based gaming [6], crime and violence [7], and worsening of externalizing MH symptoms, such as reduced concentration [8]. Despite pandemic-related increases in MH disorders and risk-taking behaviors, the temporal relationship between them and SARS-CoV-2 infection remains unclear. That is, did these behaviors influence a person’s infection susceptibility or were these behaviors more common in those infected?

A study by Wang et al [9] found that those with recent substance use disorder (SUD) diagnoses were at a higher risk for COVID-19, especially those abusing opioids. However, the relationships between COVID-19 infection and previous SUD-related behavioral problems, among other destructive behaviors, remain largely unknown. Previous research has linked deviant [10] and antisocial behaviors [11-13], aggression [10], isolation [14], and alcohol and substance use [12,13,15,16] to greater infection susceptibility across an array of infectious diseases, including swine flu, HIV, and other sexually transmitted diseases. Antisocial behaviors have also been linked to less social distancing and more social outings during the COVID-19 pandemic [17]. Because these destructive-type behaviors have been previously linked to greater infection susceptibility, we sought to study whether they would also be linked to COVID-19 infection.

The Global Appraisal of Individual Needs–Short Screener (GAIN-SS) questionnaire (Chestnut Health Systems, Bloomington, IL) has been validated to pinpoint diagnostic criteria for (1) externalizing and internalizing MH disorders, (2) substance abuse (including alcohol abuse), and (3) crime and violence in both adolescents and adults [18-23]. Questions ask for the recency of specific behaviors such as lying and gambling (externalizing behaviors), using alcohol or drugs (substance use), and selling drugs or destroying property (crime and violence). Clinically, the self-administrable GAIN-SS survey is used to screen for behavioral health disorders that would warrant more in-depth assessment or intervention. The efficacy of the GAIN-SS survey in identifying populations at risk for SUDs [24] and co-occurring substance use and MH disorders [25-27] has been validated.

In the context of COVID-19, the GAIN-SS survey has been used to investigate behavioral differences between students and nonstudents [28]. Findings demonstrated that MH issues were stable but substance use declined in youths during the pandemic. Another study found that youths in clinical settings met higher diagnostic criteria for externalizing and internalizing disorders during the pandemic than youths in the community [29]. However, research on COVID-19–related GAIN-SS behaviors across the adult population is lacking. Furthermore, the temporal relationship between the recency of GAIN-SS behaviors and subsequent SARS-CoV-2 infection remains unknown.

### Goal of This Study

In this preliminary cross-sectional study, we investigated how past GAIN-SS behaviors were related to SARS-CoV-2 infection. We tested the central hypothesis that self-reported histories of high-risk behaviors, MH disorders, and substance use issues would relate to, and predict, SARS-CoV-2 infection. This central hypothesis was structured into 3 subhypotheses, and proportion tests were used to investigate the temporality between these destructive behaviors and SARS-CoV-2 infection: (1) those with *any* history of destructive behaviors (from 1 month to >1 year ago) would have a higher proportion of positive COVID-19 tests or diagnoses when compared with those with no history of destructive behaviors (ie, “never”), (2) those reporting destructive behaviors *before* the pandemic (ie, >1 year ago) would have a higher proportion of positive COVID-19 tests or diagnoses when compared with those with no history of destructive behaviors (ie, “never”), and (3) those reporting destructive behaviors before the pandemic would have a higher proportion of positive COVID-19 tests or diagnoses when compared with all other response types (ie, between 1 month and 1 year ago and “never”; see the *Statistical Analysis* section under *Methods* for details). To further test these subhypotheses, we applied multivariable logistic regression (MVLR) with iterative downsampling to investigate the efficacy of GAIN-SS behaviors in discriminating participants with and without self-reported COVID-19. Together, the presented results implicate high-risk and destructive behaviors in SARS-CoV-2 infection and suggest that increased public messaging (eg, enforcing mask wearing) at entertainment venues, clinics, and rehabilitation centers, in addition to clinical and rehabilitation-related behavioral interventions, may be important when managing similar pandemics.

## Methods

### Participant Recruitment

The participant recruitment procedure was first detailed in Bari et al [30]. Questionnaire responses were collected between the end of February 2021 and the beginning of March 2021, approximately 1 year following the official COVID-19 pandemic declaration in the United States (March 11, 2020) [31]. Participants between the ages of 18 and 70 years were recruited by Gold Research Inc (San Antonio, Texas) using multiple methods such as (1) by invitation only using customer databases from large companies that participate in revenue-sharing agreements, (2) via social media, or (3) through direct mail. All participants were reimbursed US $10 for their participation. Recruited respondents followed a double opt-in consent procedure to participate in the study (refer to *Ethics Approval*); during this process, they also provided information about demographic attributes, including age, race, and sex. This information was used to ensure that the recruited participants represented the US census at the time of the survey (February-March 2021). During the study, the respondents were also prompted with repeated test questions to screen out those providing random and illogical responses and those showing flatline or speeder behavior. Data from those flagged as nonadherers were removed**.** To ensure adequate samples of participants with MH conditions, Gold Research oversampled 15% (7500/50,000) of the sample for MH conditions. Gold Research reported that >50,000 respondents were contacted to complete the questionnaire. Gold Research estimated that of the 50,000 participants, >37,500 (>75%) either did not respond or declined participation. Of the remaining 25% (12,500/50,000) who clicked on the survey link, >50% (>6250/12,500) did not fully complete the questionnaire. Of the >48% (≥6000/12,500) of participants who completed the survey, those who did not clear data integrity assessments were omitted. The participants meeting quality assurance procedures (including survey completion) were selected, with a limit of 500 to 520 total participants. Eligible participants were required to be between the ages of 18 and 70 years at the time of the survey, to be able to comprehend the English language, and to have access to an electronic device (eg, laptop or cell phone). The participants provided informed consent as described in *Ethics Approval*.

### Ethics Approval

All the participants provided informed consent, including for their primary participation in the study and the secondary use of their anonymized, deidentified data (ie, all identifying information was removed by Gold Research Inc before retrieval by the research group) in secondary analyses (refer to the consent prompts given in the next paragraph). The study was approved by Northwestern University’s institutional review board and was in accordance with the Declaration of Helsinki (approval number: STU00213665).

During initial recruitment, the participants were presented with the following:

Gold Research Inc., a national market research firm and its client, Northwestern University, request your participation in this study of emotional health. We will be evaluating how different emotions and experiences are connected and may relate to our emotional health. The information you provide will be kept confidential, coded to be anonymous so it cannot be connected back to you and will be used only for research purposes. Researchers will not be able to contact you or restudy you after this survey. We will not share your information with any other third party. We will also not use your information to identify you individually or use your responses to market or sell other services or products to you. As part of this effort, you will not be asked to provide any personal identifiers such as your name, email, phone number, address, or social media handles. A unique identifier will be generated for you and each survey participant to enhance privacy. As part of the survey process, we will be able to tell if you completed the survey, but we will not be able to tell which answers were yours. For this study, we are going to ask you some questions about yourself and how much you like or dislike a set of pictures. You may discontinue this study at any time. We appreciate your help with this study, given the serious challenges facing many people regarding emotional health at this time. We thank you in advance.

*1. Accept*

*2. Decline*

If the participants responded with “Accept,” they were sent a second communication:

Thank you for participating in our survey. All responses during this survey are anonymous and confidential. We will be able to tell if you completed the survey, but we will not be able to tell which answers were yours. In this study, we aim to understand how different emotions and experiences relate to visual processing.

We are going to:

*Ask you some questions about yourself

*Have you rate how much you like or dislike a set of pictures

For this study, your identity is protected and your answers are anonymous and confidential. Press “Next” to proceed.

The survey then commenced if the participants pressed “Next.”

### Data Quality Assurance

Data from 506 participants (age: median 44, IQR 30-59 years) passed Gold Research’s integrity assessment (refer to *Participant Recruitment*) and were then anonymized and sent to the research team. The data were further checked for quality and assessed against three exclusion criteria: (1) participants showed minimal variance in a picture rating task (ie, all pictures were rated the same, or ratings varied only by 1 point; resulted in the removal of 16/506, 3.2% participants [data not described here]); (2) participants indicated they had ≥10 clinician-diagnosed conditions (resulted in the removal of an additional 118/506, 23.3% participants; conditions described in Figure S1 in Multimedia Appendix 1), and (3) if *both* education level and years of education did not match *and* if they completed the questionnaire in <500 seconds (resulted in removal of an additional 6/506, 1.2% participants). From these procedures, 72.3% (366/506) of participants were cleared for statistical analysis (the unscored, uncoded data set can be found in Multimedia Appendix 2).

### Sample Size Calculation

At the time of the survey, 10% of the participants were expected to report having had a positive COVID-19 test (referred to as *test+*) or a positive COVID-19 diagnosis (referred to as *diagnosis*). Formal power analysis for a 2-sample proportion test revealed an estimated power of 0.986 when comparing the group that responded “yes” with the group that responded “no” to *test+* (*test+* sample size=36, no *test+* sample size=330; α=.05, hypothetical proportion of *test+* sample=0.8, hypothetical proportion of no *test+* sample=0.5) and a power of 0.982 when comparing the group that responded “yes” with the group that responded “no” to *diagnosis* (*diagnosis* sample size=34, no *diagnosis* sample size=332; α=.05, hypothetical proportion of *diagnosis* sample=0.8, hypothetical proportion of no *diagnosis* sample=0.5).

### The Questionnaire

The participants were asked to report their age, gender, ethnicity, handedness, annual household income, employment status, level of education, and years of schooling (Table S1 in Multimedia Appendix 1). They were asked to report whether they ever tested positive for COVID-19 (“yes” or “no”; *test+*) and whether they were ever diagnosed with COVID-19 by a medical professional (“yes” or “no”; *diagnosis*). The participants were 57.9% (212/366) female, 66.9% (245/366) White, 81.9% (300/366) right-handed, and 42.6% (156/366) employed full-time, and 28.7% (105/366) reported some level of college education (mean years of school 13; Table S1 in Multimedia Appendix 1), approximating national averages for these measures at the time of the survey. Of the 366 participants, 36 (9.8%) reported “yes” to *test+* and 34 (9.3%) reported “yes” to *diagnosis*, resembling national averages reported by the Centers for Disease and Control at the time of the survey. A total of 7.1% (26/366) of participants reported “yes” to both *test+* and *diagnosis.*

The participants also completed the GAIN-SS questionnaire (described in the *GAIN-SS Questionnaire and Scoring* section) [18].

### GAIN-SS Questionnaire and Scoring

The GAIN-SS questionnaire takes 3 to 5 minutes to complete and is designed to flag MH problems qualifying as (1) externalizing (eg, bullying and gambling) and internalizing (eg, fear and depression) [32], (2) substance abuse, and (3) crime and violence. We limited the GAIN-SS questionnaire to include 3 of the 4 categories to shorten the length of the overall survey: externalizing MH disorders (7 questions), substance abuse disorders (8 questions), and crime and violence problems (5 questions). All 20 questions, their abbreviated forms used hereafter, and their respective categories are outlined in Table 1.

The GAIN-SS question responses follow 2 formats. One format (ie, for externalizing behaviors) assesses whether the individual never experienced the behavior (“0”) or experienced ≥2 events over 1 of 4 time blocks: “1”=experienced the behavior >1 year ago, “2”=experienced the behavior 4 to 12 months ago, “3”=experienced the behavior 2 to 3 months ago, and “4”=experienced the behavior in the past month. The other format (ie, for substance abuse and crime and violence) asks when the participants last experienced a behavior using the same time blocks (refer to “0-4” in the previous sentence). Scores were obtained for each of the 3 questionnaire categories by counting the number of times the participants responded with a “2,” “3,” or “4” for all questions in a category; responses of “0” and “1” were not included in the count. For example, a participant with four “0” responses, one “1” response, and two “3” responses would have a final score of 2 (ie, only the two “3” responses were counted). A final externalizing score of 0 would indicate that the participant is unlikely to have a diagnosis, a score of 1 to 2 indicates a moderate likelihood of diagnosis, and a score of ≥3 indicates a high likelihood of diagnosis.

**Table 1:** Description of the Global Appraisal of Individual Needs–Short Screener (GAIN-SS) questions.

Q^a^	Abbreviated	GAIN-SS category	Question preamble	Full question
Q1	Lying	Externalizing	When was the last time that you did the following things two or more times?	Lied or conned to get things you wanted or to avoid having to do something
Q2	Attention	Externalizing	When was the last time that you did the following things two or more times?	Had a hard time paying attention at school, work, or home
Q3	Listening	Externalizing	When was the last time that you did the following things two or more times?	Had a hard time listening to instructions at school, work, or home
Q4	Waiting	Externalizing	When was the last time that you did the following things two or more times?	Had a hard time waiting for your turn
Q5	Bullying	Externalizing	When was the last time that you did the following things two or more times?	Were a bully or threatened other people
Q6	Fighting	Externalizing	When was the last time that you did the following things two or more times?	Started physical fights with other people
Q7	Gambling	Externalizing	When was the last time that you did the following things two or more times?	Tried to win back your gambling losses by going back another day
Q8	Alcohol	Substance abuse	When was the last time that...	…you used alcohol weekly or more often?
Q9	Cannabis	Substance abuse	When was the last time that...	…you used cannabis weekly or more often?
Q10	Opioid	Substance abuse	When was the last time that...	…you used heroin, fentanyl, or other opiates?
Q11	Stimulant	Substance abuse	When was the last time that...	…you used a stimulant like cocaine or meth?
Q12	Time	Substance abuse	When was the last time that...	…you spent a lot of time either getting alcohol or other drugs, using alcohol or other drugs, or recovering from the effects of alcohol or other drugs (e.g., feeling sick)?
Q13	Trouble	Substance abuse	When was the last time that...	…you kept using alcohol or other drugs even though it was causing social problems, leading to fights, or getting you into trouble with other people?
Q14	Isolation	Substance abuse	When was the last time that...	…your use of alcohol or other drugs caused you to give up or reduce your involvement in activities at work, school, home, or social events?
Q15	Symptoms	Substance abuse	When was the last time that...	…you had withdrawal problems from alcohol or other drugs like shaky hands, throwing up, having trouble sitting still or sleeping, or you used any alcohol or other drugs to stop being sick or avoid withdrawal problems?
Q16	Violence	Crime and violence	When was the last time that you...	…had a disagreement in which you pushed, grabbed, or shoved someone?
Q17	Stealing	Crime and violence	When was the last time that you...	…took something from a store without paying for it?
Q18	Selling drugs	Crime and violence	When was the last time that you...	…sold, distributed, or helped to make illegal drugs?
Q19	DUI^b^	Crime and violence	When was the last time that you...	…drove a vehicle while under the influence of alcohol or illegal drugs?
Q20	Destruction	Crime and violence	When was the last time that you...	…purposely damaged or destroyed property that did not belong to you?

^a^Q: question.

^b^DUI: driving under the influence.

### Statistical Analyses

#### Analysis of Demographics and GAIN-SS by Self-reported COVID-19

Demographic variables (Table S1 in Multimedia Appendix 1), GAIN-SS scores, and individual GAIN-SS question responses were assessed for differences between those who responded “yes” and those who responded “no” to *test+*, *diagnosis,* or both using the Wilcoxon rank sum test [33]. Significant categorical demographic variables were further assessed for distribution equality using the Kolmogorov-Smirnov test (α=.05) [34]. Results with significant *P* values (α=.05) were corrected for multiple comparisons using the Benjamini-Hochberg procedure (reported as *Q* values) [35]. Box plots were generated for significant results (*Q* value<0.05).

#### GAIN-SS Coding

GAIN-SS responses were coded to test 3 subhypotheses, all of which evaluated whether the participants with prior destructive and high-risk behaviors had higher proportions of self-reported COVID-19. The subhypotheses are detailed in the following section and in Table 2.

**Table 2:** Description of Global Appraisal of Individual Needs–Short Screener question coding.

Subhypothesis name	Subhypothesis description	Coding^a^
*never vs. anytime*	Participants with a prior history of destructive or risky behaviors report a higher proportion of COVID-19 compared with those never exhibiting behaviors	(“0,” never=*0*), (“1,” >1 year ago=*1*), (“2,” 4 to 12 months ago=*1*), (“3,” 2 to 3 months ago=*1*), and (“4,” past month=*1*)
*never vs. >1 year*	Participants reporting destructive or risky behaviors ≥1years ago report a higher proportion of COVID-19 compared with those never exhibiting behaviors	(“0,” never=*0*), (“1,” >1 year ago=*1*), and (“4,” “3,” and “2” dropped)
*anytime and never vs. >1 year*	Participants reporting destructive or risky behaviors ≥1 years ago report a higher proportion of COVID-19 compared with those never, or more recently, exhibiting behaviors	(“1,” >1 year ago=*1*), (“0,” never=*0*), (“2,” 4 to 12 months ago=*0*), (“3,” 2 to 3 months ago=*0*), and (“4,” past month=*0*)

^a^Quotation marks (“ ”) indicate the numerical Global Appraisal of Individual Needs–Short Screener response, followed by the definition associated with the numerical response and then the coding of the response in italics.

#### GAIN-SS Proportion Tests by Self-reported COVID-19

The first subhypothesis included *all* GAIN-SS responses (“0-4”). It tested whether the participants who exhibited GAIN-SS behaviors at any prior time (past month, 2 to 3 months ago, 4 to 12 months ago, and >1 year ago) were more likely to respond “yes” to having had a positive COVID-19 test (*test+*) or *diagnosis* than the participants who responded “no” to *test+* or *diagnosis.* The response to “never” was coded as 0 and all other responses (past month, 2 to 3 months, 4 to 12 months, and >1 years) were coded as 1. This subhypothesis is referred to as *never vs. anytime* henceforth.

The second subhypothesis *excluded* the participants who exhibited GAIN-SS behaviors more recently (ie, past month to 12 months ago). To investigate whether the participants who exhibited GAIN-SS–related behaviors >1 year ago (ie, before the pandemic) were more likely to report “yes” for COVID-19, “never” was coded as 0, and “1+ years ago” was coded as 1. This subhypothesis is referred to as *never vs*. *>1 year* henceforth.

The third subhypothesis *included* the participants who exhibited GAIN-SS behaviors more recently (ie, past month to 12 months ago). It tested whether the participants who exhibited GAIN-SS–related behaviors >1 year ago were more likely to report “yes” for COVID-19. For this case, “never” and more recent responses (ie, past month, 2 to 3 months, and 4 to 12 months) were coded as 0, and “1+ years ago” was coded as 1. This subhypothesis is referred to as *anytime and never vs. >1 year* henceforth.

For each subhypothesis, proportion tests were performed to obtain both nondirectional and directional *P* values (α=.05). For the case where the participants who exhibited GAIN-SS behaviors had a higher proportion of “yes” responses to *test+* or *diagnosis* (*P* [YES>NO]), a Benjamini-Hochberg correction was performed to obtain *Q* values (*Q* [YES>NO]). GAIN-SS questions implicated with higher proportions of “yes” responses to *test+* and *diagnosis* (*Q* [YES>NO] <0.05) were further analyzed using MVLR. Refer to Multimedia Appendix 1 for a more complete description of all 3 subhypotheses.

#### MVLR and Iterative Downsampling: Using GAIN-SS to Predict COVID-19

MVLR was performed for each of the 3 subhypotheses using demographic variables that significantly differed between those who responded “yes” and those who responded “no” to *test+* and *diagnosis*, as well as significant GAIN-SS behaviors as determined from proportion tests (refer to the previous section). Self-reported COVID-19 (where “yes”=1 and “no”=0) was a binary dependent variable, demographics were covariates (age, income, and education level), and GAIN-SS responses (ie, responses that significantly differed in each corresponding proportion test) were the independent variables. Because more recent time point responses were dropped when testing *never vs. >1 year*, resulting in different response distributions to “1+ years ago” for each question, GAIN-SS behaviors were analyzed independently for this subhypothesis.

Because the percentage of participants who responded “yes” to *test+, diagnosis, or both* (33-37/366, 9%-10%) was much smaller than the percentage of those who responded “no” (329-333/366, 89.9%-91%), the following procedures were performed to avoid overfitting the MVLR models to the majority class (ie, participants who responded “no” to *test+* and *diagnosis*). Data from the majority class were randomly downsampled 1000 times to match the sample size of those who self-reported COVID-19 (“yes”: 36/366, 9.8% for *test+* model and 34/366, 9.3% for *diagnosis* model). Downsampling was iterated 1000 times to obtain better estimates of the measures reported. MVLR was run at each iteration for the downsampled data to obtain model accuracy, root mean square error, mean absolute error, area under the receiver operating characteristic curve (AUROC), sensitivity, specificity, positive predictive value (PPV), and negative predictive value (NPV). Average measures across all iterations were reported. Crude and adjusted odds ratios with respective logit estimates, *z* scores, *P* values, and SEs from 1 iteration were also reported for representative models. Please note that, given the sample size of the minority class, MVLR was run without separate training and test sets. The accuracy was computed by dividing the number of times the model correctly determined the binary outcome by the size of the downsampled data.

## Results

### Demographic Variables Varied by Self-reported COVID-19

**Figure 1:**
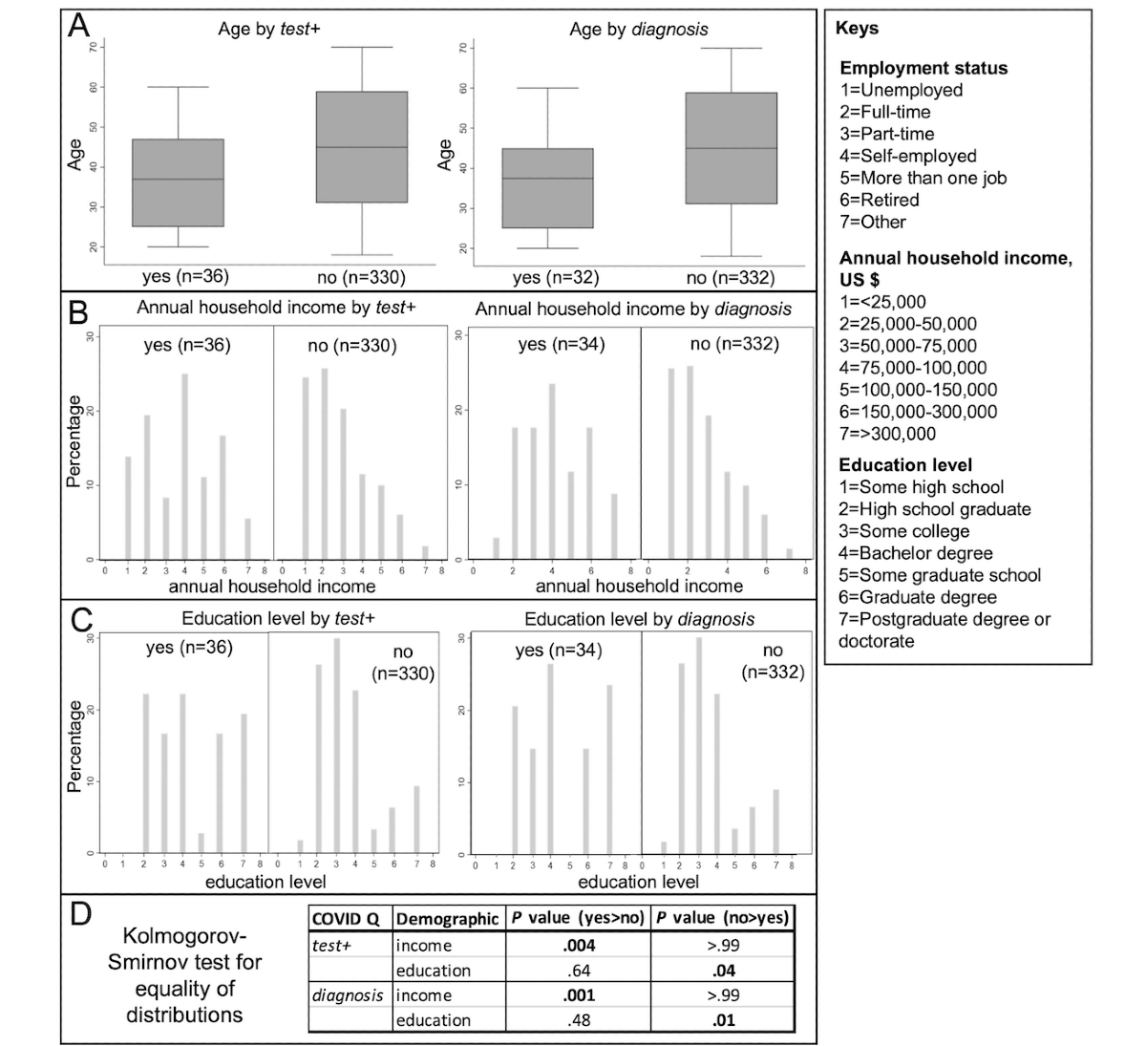
Demographic trends across those who reported “yes” and those who reported “no” to COVID-19 *test+* and *diagnosis*. (A) Age differences by *test+* and *diagnosis*. (B) Household income differences by *test+* and *diagnosis*. (C) Education level differences by *test+* and *diagnosis*. (D) Kolmogorov-Smirnov test for distribution equality; *P*<.05 is considered significant (bolded).

Age and income significantly varied by *test+*, whereas age, income, and education level significantly varied by *diagnosis* (*Q*=0.024 for *test+* and *Q*=0.020 for *diagnosis*; Figure 1, Table 3; all results reported in Table S2 in Multimedia Appendix 1). The participants who responded “yes” to *test+* and *diagnosis* were, on average, younger than those who responded “no” (Figure 1A). Specifically, middle-aged adults more frequently reported “yes” to *test+* (median 37, IQR 25-47 years) and *diagnosis* (median 37.5, IQR 25-45 years), as compared with those who responded “no” (median 45, IQR 31-59 years). The participants who responded “no” to *test+* or *diagnosis* fell on a left-skewed distribution, implying a higher percentage of low self-reported annual household income as compared with the participants who responded “yes” (Wilcoxon rank sum test *Q*=0.02 for *test+* and *Q*<0.001 for *diagnosis*; Figure 1B). Those who responded “yes” to *test+* or *diagnosis* exhibited a bimodal distribution of education level, whereas the distribution for “no” responses was skewed left (Wilcoxon rank sum test *P*=.02; *Q*=0.08 for *test+* and *Q*=0.04 for *diagnosis*; Figure 1C). The Kolmogorov-Smirnov test confirmed the differences between the “yes” and “no” distributions (Figure 1D). Income distributions were different between those who responded “yes” and those who responded “no” to *test+* (*P*=.004) and *diagnosis* (*P*=.001); specifically, more persons responded “yes” to COVID-19 in the higher income categories. Education level distributions were different between those who responded “yes” and those who responded “no” to *test+* (*P*=.04) and *diagnosis* (*P*=.01); specifically, more persons with higher reported education responded “yes” to COVID-19.

**Table 3:** Wilcoxon rank sum test results.^a^

Category			COVID-19 Q^b^		Variable		*P* value^c^		*Q* value^d^
**Demographics**									
	*N/A* ^e^	*Test+*		*Age*		*.006*		*0.02*	
	*N/A*	*Diagnosis*		*Age*		*.004*		*0.02*	
	N/A	Diagnosis		Employment		.04		0.20	
	*N/A*	*Test+*		*Income*		*.004*		*0.02*	
	*N/A*	*Diagnosis*		*Income*		*<.001*		*<0.001*	
	N/A	Test+		Education level		.02		.008	
	*N/A*	*Diagnosis*		*Education level*		*.009*		*0.04*	
**GAIN-SS^f^ categories and questions**									
	Externalizing behavior	Test+		Overall		.002		N/A	
	Externalizing behavior	Test+		Attention (Q2)		.03		0.15	
	Externalizing behavior	Test+		Bullying (Q5)		.04		0.15	
	Externalizing behavior	Test+		Fighting (Q6)		.04		0.15	
	*Externalizing behavior*	*Test+*		*Gambling (Q7)*		*<.001*		*0.004*	
	Substance abuse	Test+		Stimulant (Q4)		.01		0.09	
	Crime and violence	Test+		Overall		<.001		N/A	
	*Crime and violence*	*Test+*		*Violence (Q16)*		*.006*		*0.02*	
	*Crime and violence*	*Test+*		*Selling drugs (Q18)*		*<.001*		*0.002*	
	*Crime and violence*	*Test+*		*Destruction (Q20)*		*<.001*		*<0.001*	
	Externalizing behavior	Diagnosis		Overall		<.001		N/A	
	*Externalizing behavior*	*Diagnosis*		*Lying (Q1)*		*.003*		*0.01*	
	*Externalizing behavior*	*Diagnosis*		*Attention (Q2)*		*.005*		*0.01*	
	*Externalizing behavior*	*Diagnosis*		*Listening (Q3)*		*.009*		*0.02*	
	*Externalizing behavior*	*Diagnosis*		*Waiting (Q4)*		*.03*		*0.03*	
	*Externalizing behavior*	*Diagnosis*		*Bullying (Q5)*		*<.001*		*<0.001*	
	*Externalizing behavior*	*Diagnosis*		*Fighting (Q6)*		*.003*		*0.01*	
	*Externalizing behavior*	*Diagnosis*		*Gambling (Q7)*		*<.001*		*<0.001*	
	Substance abuse	Diagnosis		Overall		.004		N/A	
	Substance abuse	Diagnosis		Alcohol (Q8)		.04		0.09	
	*Substance abuse*	*Diagnosis*		*Opioid (Q10)*		*.009*		*0.04*	
	*Substance abuse*	*Diagnosis*		*Stimulant (Q4)*		*.002*		*0.01*	
	*Substance abuse*	*Diagnosis*		*Time (Q12)*		*.004*		*0.03*	
	*Substance abuse*	*Diagnosis*		*Trouble (Q13)*		*.01*		*0.04*	
	*Substance abuse*	*Diagnosis*		*Isolation (Q14)*		*.02*		*0.045*	
	*Substance abuse*	*Diagnosis*		*Symptoms (Q15)*		*.01*		*0.04*	
	Crime and violence	Diagnosis		Overall		<.001		N/A	
	*Crime and violence*	*Diagnosis*		*Violence (Q16)*		*<.001*		*0.002*	
	*Crime and violence*	*Diagnosis*		*Selling drugs (Q18)*		*<.001*		*0.001*	
	Crime and violence	Diagnosis		DUI^g^ (Q19)		.04		0.08	
	*Crime and violence*	*Diagnosis*		*Destruction (Q20)*		*<.001*		*<0.001*	

^a^Italicized rows indicate *Q*<0.05.

^b^Q: question.

^c^*P* value is determined using a Wilcoxon rank sum test where “Variable” is tested for differences between “COVID-19 Q.”

^d^*Q*-value is the Benjamini-Hochberg corrected *P* value. Italicized values indicate that the reported values passed a threshold for significance.

^e^N/A: not applicable.

^f^GAIN-SS: Global Appraisal of Individual Needs–Short Screener.

^g^DUI: driving under the influence.

### GAIN-SS Scores and Question Responses Varied by Self-reported COVID-19

GAIN-SS scores (externalizing, substance abuse, and crime and violence) were higher for those who reported “yes” to *test+* (externalizing *P*=.002; crime and violence *P*<.001) or *diagnosis* (externalizing *P*<.001; substance abuse *P*=.004; and crime and violence *P*<.001; Wilcoxon rank sum test; Table 3). Responses to specific questions varied by *test+* (*Q*<0.05; Wilcoxon rank sum test followed by Benjamini-Hochberg correction): gambling (*Q*=0.004), violence (*Q*=0.02), selling drugs (*Q*=0.002), and destruction of property (*Q*=<0.001; Table 3 and Figure S2 in Multimedia Appendix 1). Responses to a larger set of questions (18 out of 20 total questions) varied by *diagnosis* (refer to Table 3 for all *Q*<0.05; Figure S2 in Multimedia Appendix 1). The complete set of results are reported in Table S3 in Multimedia Appendix 1.

### Participants Who Exhibited GAIN-SS Behaviors Reported Higher Proportions of COVID-19

Testing the 3 subhypotheses produced multiple outcomes. The coding procedure for each respective subhypothesis can be found in Table 4.

For *never vs anytime*, there were 23 significant proportion test results (refer to Table 5 for all *Q*<0.05), where participants who exhibited GAIN-SS behaviors at any prior time (ie, past month, 2 to 3 months ago, 4 to 12 months ago, and >1 year ago) had a higher proportion of “yes” responses than “no” responses to *test+* and *diagnosis* compared with those responding “no” (see Table 5 for all *Q* [YES>NO]). Most results (17/23, 74%) involved *diagnosis*.

For *never vs. >1 year*, there were 8 significant results (refer to Table 6 for all *Q*<0.05), where participants who exhibited GAIN-SS behaviors >1 year ago had a higher proportion of “yes” responses than “no” responses to *test+* and *diagnosis* compared with those responding “no” (refer to Table 6 for all *Q* [YES>NO]).

For *anytime and never vs. >1 year*, there were 4 significant results (refer to Table 7 for all *Q*<0.05), where the participants who exhibited GAIN-SS behaviors >1 year ago had a higher proportion of “yes” responses than “no” responses to *test+* and *diagnosis* when compared with those responding “no” (refer to Table 7 for all *Q* [YES>NO]).

**Table 4:** The coding criteria for each proportion test hypothesis: (1) never versus anytime, (2) never versus >1 year ago, and (3) anytime and never versus >1 year ago. “Yes” responses to *test+* and *diagnosis* were coded as 0, and “no” responses to *test+* and *diagnosis* were coded as 1.

Test	Coding
*never versus anytime*	(0, NEVER=0), (1, >1 YEAR=1), (2, 4 to 12 months=1), (3, 2 to 3 months=1), (4, past month=1)
*never versus >1 year*	(0, NEVER=0), (1, >1 YEAR=1), (4, 3, and 2 dropped)
*anytime and never versus >1 year*	(1, >1 YEAR=1), (0, NEVER=0), (2, 4 to 12 months=0), (3, 2 to 3 months=0), (4, past month=0)
no COVID-19 (test or diagnosis)	0
yes COVID-19 (test or diagnosis)	1

**Table 5:** Proportion test results for hypothesis comparing the participants who never reported a given destructive behavior versus those who reported a destructive behavior at any time.

COVID-19 Q^a^, GAIN-SS^b^ category, and GAIN-SS Q				*P* value (nondirectional)^c^		*P* value (no>yes)^d^		*P* value (yes>no)^e^		*Q* value (yes>no)^f^
**Test+**										
	**Externalizing**									
		Lying (Q1)	.22		.89		.11		0.11	
		*Attention (Q2)* ^g^	*.004*		*>.99*		*.002*		*0.01*	
		Listening (Q3)	.05		.97		.03		0.08	
		Waiting (Q4)	.08		.96		.04		0.08	
		*Bullying (Q5)*	*.02*		*>.99*		*.01*		*0.048*	
		Fighting (Q6)	.05		.97		.03		0.08	
		*Gambling (Q7)*	*<.001*		*>.99*		*<.001*		*<0.001*	
	**Substance abuse**									
		Alcohol (Q1)	.16		.92		.08		0.16	
		Cannabis (Q2)	.51		.74		.26		0.26	
		Opioid (Q3)	.09		.96		.04		0.15	
		Stimulant (Q4)	.02		>.99		.008		0.06	
		Time (Q5)	.07		.97		.03		0.15	
		Trouble (Q6)	.10		.95		.05		0.15	
		Isolation (Q7)	.10		.95		.05		0.15	
		Symptoms (Q8)	.09		.96		.04		0.15	
	**Crime and violence**									
		*Violence (Q1)*	*.01*		*>.99*		*.007*		*0.02*	
		Stealing (Q2)	.33		.84		.16		0.18	
		*Selling drugs (Q3)*	*<.001*		*>.99*		*<.001*		*<0.001*	
		DUI^h^ (Q4)	.36		.82		.18		0.18	
		*Destruction (Q5)*	*<.001*		*>.99*		*<.001*		*<0.001*	
**Diagnosis**										
	**Externalizing**									
		*Lying (Q1)*	*.002*		*>.99*		*.001*		*0.004*	
		*Attention (Q2)*	*<.001*		*>.99*		*<.001*		*<0.001*	
		*Listening (Q3)*	*.007*		*>.99*		*.004*		*0.007*	
		*Waiting (Q4)*	*.01*		*>.99*		*.007*		*0.007*	
		*Bullying (Q5)*	*<.001*		*>.99*		*<.001*		*<0.001*	
		*Fighting (Q6)*	*.003*		*>.99*		*.001*		*0.004*	
		*Gambling (Q7)*	*<.001*		*>.99*		*<.001*		*<0.001*	
	**Substance abuse**									
		*Alcohol (Q1)*	*.01*		*>.99*		*.005*		*0.01*	
		Cannabis (Q2)	.18		.91		.09		0.09	
		*Opioid (Q3)*	*.004*		*>.99*		*.002*		*0.01*	
		*Stimulant (Q4)*	*.002*		*>.99*		*.001*		*0.008*	
		*Time (Q5)*	*.004*		*>.99*		*.002*		*0.01*	
		*Trouble (Q6)*	*.006*		*>.99*		*.003*		*0.01*	
		*Isolation (Q7)*	*.006*		*>.99*		*.003*		*0.01*	
		*Symptoms (Q8)*	*.005*		*>.99*		*.003*		*0.01*	
	**Crime and violence**									
		*Violence (Q1)*	*.002*		*>.99*		*<.001*		*0.0021*	
		Stealing (Q2)	.10		.95		.05		0.06	
		*Selling drugs (Q3)*	*<.001*		*>.99*		*<.001*		*<0.001*	
		DUI (Q4)	.12		.94		.06		0.06	
		*Destruction (Q5)*	*<.001*		*>.99*		*<.001*		*<0.001*	

^a^Q: question.

^b^GAIN-SS: Global Appraisal of Individual Needs–Short Screener.

^c^*P* value when conducting a nondirectional proportion test.

^d^Directional proportion test *P* value for the case where there was a higher proportion of those who did not report COVID-19.

^e^Directional proportion test *P* value for the case where there was a higher proportion of those who reported COVID-19.

^f^The Benjamini-Hochberg corrected *P* value for the case where there was a higher proportion of those who reported COVID-19.

^g^Italicized values meet significance (*Q*<0.05).

^h^DUI: driving under the influence.

**Table 6:** Proportion test results for hypothesis comparing the participants who never reported a given destructive behavior with those who reported a destructive behavior >1 year ago.

COVID-19 Q^a^, GAIN-SS^b^ category, and GAIN-SS Q			*P* value (nondirectional)^c^	*P* value (no>yes)^d^	*P* value (yes>no)^e^	*Q* value (yes>no)^f^
**Test+**						
	**Externalizing**					
		Lying (Q1)	.96	.52	.48	0.78
		Attention (Q2)	.04	.98	.02	0.11
		Listening (Q3)	.06	.97	.03	0.11
		Waiting (Q4)	.88	.56	.44	0.78
		Bullying (Q5)	.05	.97	.03	0.11
		Fighting (Q6)	.43	.22	.78	0.78
		*Gambling (Q7)* ^g^	*<.001*	*>.99*	*<.001*	*<0.001*
	**Substance abuse**					
		Alcohol (Q1)	.56	.72	.28	0.58
		Cannabis (Q2)	.42	.79	.21	0.58
		Opioid (Q3)	.29	.86	.14	0.58
		Stimulant (Q4)	.84	.42	.58	0.58
		Time (Q5)	.46	.77	.23	0.58
		Trouble (Q6)	.89	.56	.44	0.58
		Isolation (Q7)	.22	.89	.11	0.58
		Symptoms (Q8)	.99	.49	.51	0.58
	**Crime and violence**					
		Violence (Q1)	.79	.39	.61	0.97
		Stealing (Q2)	.86	.43	.57	0.97
		*Selling drugs (Q3)*	*.001*	*>.99*	*<.001*	*0.004*
		DUI^h^ (Q4)	.06	.03	.97	0.97
		Destruction (Q5)	.10	.95	.05	0.20
**Diagnosis**						
	**Externalizing**					
		Lying (Q1)	.06	.97	.03	0.11
		*Attention (Q2)*	*.002*	*>.99*	*<.001*	*0.005*
		Listening (Q3)	.20	.90	.10	0.20
		Waiting (Q4)	.14	.93	.07	0.20
		*Bullying (Q5)*	*.001*	*>.99*	*<.001*	*0.004*
		Fighting (Q6)	.86	.57	.43	0.43
		*Gambling (Q7)*	*<.001*	*>.99*	*<.001*	*<0.001*
	**Substance abuse**					
		Alcohol (Q1)	.19	.90	.10	0.38
		Cannabis (Q2)	.11	.95	.05	0.33
		Opioid (Q3)	.18	.91	.09	0.38
		Stimulant (Q4)	.95	.52	.48	0.48
		Time (Q5)	.61	.69	.31	0.48
		Trouble (Q6)	.65	.68	.32	0.48
		*Isolation (Q7)*	*.007*	*>.99*	*.003*	*0.03*
		Symptoms (Q8)	.09	.96	.04	0.31
	**Crime and violence**					
		Violence (Q1)	.5	.71	.29	0.57
		Stealing (Q2)	.57	.71	.29	0.57
		*Selling drugs (Q3)*	*<.001*	*>.99*	*<.001*	*0.002*
		DUI (Q4)	.23	.12	.88	0.88
		*Destruction (Q5)*	*.007*	*>.99*	*.003*	*0.01*

^a^Q: question.

^b^GAIN-SS: Global Appraisal of Individual Needs–Short Screener.

^c^*P* value when conducting a nondirectional proportion test.

^d^Directional proportion test *P* value for the case where there was a higher proportion of those who did not report COVID-19.

^e^Directional proportion test *P* value for the case where there was a higher proportion of those who reported COVID-19.

^f^The Benjamini-Hochberg corrected *P* value for the case where there was a higher proportion of those who reported COVID-19.

^g^Italicized values meet significance (*Q*<0.05).

^h^DUI: driving under the influence.

**Table 7:** Proportion test results for hypothesis comparing the participants who never reported a given destructive behavior or those who reported a destructive behavior at any time with those who reported a destructive behavior >1 year ago.

COVID-19 Q^a^, GAIN-SS^b^ category, and GAIN-SS Q			*P* value (nondirectional)^c^	*P* value (no>yes)^d^	*P* value (yes>no)^e^	*Q* value (yes>no)^f^
**Test+**						
	**Externalizing**					
		Lying (Q1)	.61	.30	.70	0.86
		Attention (Q2)	.43	.78	.22	0.86
		Listening (Q3)	.14	.93	.07	0.35
		Waiting (Q4)	.67	.34	.66	0.86
		Bullying (Q5)	.11	.94	.06	0.34
		Fighting (Q6)	.29	.14	.86	0.86
		*Gambling (Q7)* ^g^	*<.001*	*>.99*	*<.001*	*0.002*
	**Substance abuse**					
		Alcohol (Q1)	.97	.52	.48	0.76
		Cannabis (Q2)	.47	.77	.24	0.76
		Opioid (Q3)	.37	.81	.19	0.76
		Stimulant (Q4)	.47	.24	.76	0.76
		Time (Q5)	.69	.65	.35	0.76
		Trouble (Q6)	.88	.44	.56	0.76
		Isolation (Q7)	.32	.84	.16	0.76
		Symptoms (Q8)	.80	.40	.60	0.76
	**Crime and violence**					
		Violence (Q1)	.35	.17	.83	0.99
		Stealing (Q2)	.67	.34	.66	0.99
		*Selling drugs (Q3)*	*.007*	*>.99*	*.003*	*0.02*
		DUI^h^ (Q4)	.02	.01	.99	0.99
		Destruction (Q5)	.32	.84	.16	0.65
**Diagnosis**						
	**Externalizing**					
		Lying (Q1)	.55	.72	.28	0.61
		Attention (Q2)	.15	.93	.07	0.37
		Listening (Q3)	.84	.58	.42	0.61
		Waiting (Q4)	.44	.78	.22	0.61
		Bullying (Q5)	.02	.99	.01	0.06
		Fighting (Q6)	.77	.39	.61	0.61
		*Gambling (Q7)*	*.004*	*>.99*	*.002*	*0.01*
	**Substance abuse**					
		Alcohol (Q1)	.85	.57	.42	0.73
		Cannabis (Q2)	.16	.92	.08	0.55
		Opioid (Q3)	.32	.84	.16	0.73
		Stimulant (Q4)	.55	.27	.73	0.73
		Time (Q5)	.92	.46	.54	0.73
		Trouble (Q6)	.97	.48	.52	0.73
		Isolation (Q7)	.02	>.99	.009	0.07
		Symptoms (Q8)	.20	.90	.10	0.59
	**Crime and violence**					
		Violence (Q1)	.77	.39	.61	0.96
		Stealing (Q2)	.81	.59	.41	0.96
		*Selling drugs (Q3)*	*.004*	*>.99*	*.002*	*0.01*
		DUI (Q4)	.09	.04	.96	0.96
		Destruction (Q5)	.07	.97	.04	0.14

^a^Q: question.

^b^GAIN-SS: Global Appraisal of Individual Needs–Short Screener.

^c^*P* value when conducting a nondirectional proportion test.

^d^Directional proportion test *P* value for the case where there was a higher proportion of those who did not report COVID-19.

^e^Directional proportion test *P* value for the case where there was a higher proportion of those who reported COVID-19.

^f^The Benjamini-Hochberg corrected *P* value for the case where there was a higher proportion of those who reported COVID-19.

^g^Italicized values meet significance (*Q*<0.05).

^h^DUI: driving under the influence.

### A Subset of GAIN-SS Behaviors Predicted Self-reported COVID-19

MVLR tested the efficacy of using GAIN-SS behaviors to predict “yes/no” responses to *test+* and *diagnosis*. MVLR models were run using significant GAIN-SS behaviors from each of the 3 proportion tests (Tables 5-7). The covariates age, income, and education level were also included in each model based on Wilcoxon rank sum test results (Table 3).

When *diagnosis* was the dependent variable, the model with the highest accuracy (accuracy=95.55%, sensitivity=95.94%, specificity=95.81%, PPV=95.87%, NPV=95.98%; AUROC=0.96) included attention problems (for subhypothesis *never vs. >1 year*; Table 8). The second highest accuracy (accuracy=95.09%, sensitivity=95.48%, specificity=95.81%, PPV=95.73%, NPV=95.55%; AUROC=0.96) resulted from a model with a large number of GAIN-SS behaviors (for subhypothesis *never vs. anytime*; Table 8).

When *test+* was the dependent variable, the model with the highest accuracy (accuracy=78.39%, sensitivity=79.99%, specificity=76.15%, PPV=77.11%, NPV=79.42%; AUROC=0.78) included gambling (for subhypothesis *never vs. >1 year*; Table 8). The second highest accuracy (accuracy: 78.31%, sensitivity=78.41%, specificity=78.34%, PPV=78.44%, NPV=78.51%; AUROC=0.78) resulted from a model including attention, bullying, gambling, violent behavior, selling drugs, and destruction of property (for subhypothesis *never vs. anytime*; Table 8).

Model accuracies ranged between 77.42% and 99.55%, where the model accuracy for predicting *diagnosis* was consistently higher. The inclusion of covariates in the model was important; however, they were not independently responsible for the high accuracies observed when GAIN-SS behaviors were also included in the model (Table S4 in Multimedia Appendix 1).

Odds ratios and related metrics can be found in Figure S3 in Multimedia Appendix 1**.**

**Table 8:** Multivariable logistic regression results.

Subhypothesis and COVID-19 question (DV^a^)		IVs^b^	Covariates	Average accuracy	SD accuracy^c^	Average RMSE^d^	Average MAE^e^	AUROC^f^	Sensitivity	Specificity	PPV^g^	NPV^h^
***never vs. anytime***												
	Test+	Attention, bullying, gambling, violence, selling drugs, destruction	Age, income, educated level	78.31	4.5	0.46	0.22	0.78	78.41	78.34	78.44	78.51
	Diagnosis	Attention, listening, waiting, bullying, fighting, gambling, opioid, stimulant, time, symptoms, violence, selling drugs, destruction, lying, alcohol, trouble, isolation	Age, income, education level	95.09	5.78	0.16	0.05	0.96	95.48	95.71	95.73	95.55
***never vs. >1 year***												
	Test+	Gambling	Age, income, education	78.39	4.88	0.46	0.22	0.78	79.99	76.15	77.11	79.42
	Test+	Selling drugs	Age, income, education	77.72	4.96	0.47	0.22	0.78	79.19	76.49	77.24	78.79
	Diagnosis	Attention	Age, income, education	95.55	5.49	0.15	0.045	0.96	95.94	95.81	95.87	95.98
	Diagnosis	Bullying	Age, income, education	83.67	5.63	0.40	0.16	0.84	83.99	83.02	83.31	83.97
	Diagnosis	Gambling	Age, income, education	83.05	5.71	0.40	0.17	0.83	84.02	82.10	82.58	83.88
	Diagnosis	Selling drugs	Age, income, education	81.3	5.19	0.43	0.19	0.81	81.66	80.88	81.12	81.64
	Diagnosis	Destruction	Age, income, education	82.75	5.86	0.41	0.17	0.83	82.83	82.52	82.67	82.92
	Diagnosis	Isolation	Age, income, education	81.82	5.27	0.42	0.18	0.81	80.56	81.94	81.75	80.99
***anytime and never vs. >1 year***												
	Test+	Gambling, selling drugs	Age, income, education	75.18	4.48	0.5	0.25	0.76	77.57	73.58	74.71	76.77
	Diagnosis	Gambling, selling drugs	Age, income, education	78.58	4.48	0.46	0.21	0.79	78.54	78.76	78.79	78.74

^a^DV: dependent variable.

^b^IV: independent variable.

^c^The standard definition of the average accuracy.

^d^RMSE: root mean square error.

^e^MAE: mean absolute error.

^f^AUROC: area under the receiver operating characteristic curve.

^g^PPV: positive predictive value.

^h^NPV: negative predictive value.

## Discussion

### Principal Findings

This study produced 3 main findings using a population sample of 366 participants (varying samples of 70 participants were included in MVLR analyses after downsampling to the minority class). First, self-reported COVID-19 was more common in younger persons with diverse income and education levels. Second, those who self-reported COVID-19 were more likely to report prior destructive and high-risk behaviors. Third, prior history of destructive and high-risk behaviors accurately modeled self-reported COVID-19. The participants who reported a history of risk-taking and destructive behaviors (in particular, gambling and drug selling) were more likely to contract SARS-CoV-2, and thus a history of risk-taking, in the absence of current risk-taking, was accurate to discriminate between participants with and participants without self-reported COVID-19. These findings support the hypothesis that prior risk-taking behaviors can predict later SARS-CoV-2 infection.

### SARS-CoV-2 Infection Is More Common in Younger Persons With Diverse Incomes and Education Levels

In our sample, age, income, and education level significantly varied by self-reported COVID-19 (*test+*/*diagnosis*=“yes/no”). Middle-aged adults (approximately between the ages of 25 and 45 years) more frequently reported “yes” to *test+* and *diagnosis* compared with older adults (aged >45 years). These results contrast with some reports of SARS-CoV-2 incidence [36] but support other studies where younger to middle-aged persons were more likely to contract SARS-CoV-2 [37]. This observation could be the result of many factors, including vaccine availability, which was initially prioritized to older adults and susceptible populations up until it was more widely available in the late winter or early spring of 2021 (around the time this survey was administered) [38]. Older adults may also be more likely to follow prevention protocols (ie, mask wearing) than younger adults with more social contacts and less feelings of vulnerability [39].

Annual household income distributions varied between those who self-reported COVID-19 and those who did not. Those who responded “yes” to *test+* and *diagnosis* displayed more Gaussian-like distributions than those who responded “no,” among whom the distributions were skewed left toward lower income levels. In our sample, SARS-CoV-2 infection occurred in persons with a wide range of income levels and did not preferentially affect those with lower or higher incomes. These findings are consistent with the divergence of findings regarding income and COVID-19 incidence in the United States. Some studies reported that individuals living in higher income households (>US $75,000 annually) [37] or counties [40] had a greater probability of contracting SARS-CoV-2 in the United States, whereas other studies reported higher SARS-CoV-2 incidence and severity in lower income households [41-46].

Education level followed a bimodal-like distribution in those who responded “yes” for COVID-19, whereas the distribution skewed left for those who responded “no.” These results suggest that education level did not predispose persons for SARS-CoV-2 infection, although the percentage of those with higher levels of education was greater among those who reported SARS-CoV-2 infection than among those who did not report SARS-CoV-2 infection. A report by Rattay et al [47] demonstrated that low education was associated with higher perceived COVID-19 severity and lower perceived probability of infection, albeit the differences were small and the authors iterated the importance of risk messaging to all persons, regardless of their education level. A UK study reported a higher risk for COVID-19 in those with the lowest education level [48], but another study in China reported a higher percentage of COVID-19 infection in those with a college education or higher level of education [49]. In general, the relationships between education level and COVID-19 susceptibility remain unclear, and there are many other confounding factors that may drive observations (age, income, etc).

### Destructive and Risk-Taking Behaviors Were More Common in Persons Who Reported COVID-19

Both overall scores (externalizing, substance abuse, and crime and violence) and individual GAIN-SS responses differed between those who responded “yes” and those who responded “no” for COVID-19. Scores were higher and individual behaviors were more frequently reported for all time blocks (as compared with never) in those who responded “yes” for COVID-19. These results suggest that those exhibiting destructive behaviors more frequently, or more recently, may be more likely to contract SARS-CoV-2.

Among destructive and risk-taking behaviors, those who reported COVID-19 had higher proportions of gambling and drug selling behaviors across all 3 subhypotheses (Tables 5-7). These observations support multiple studies that highlight increased gaming, web-based shopping, and web-based gambling behaviors during the SARS-CoV-2 pandemic [6,50,51]. However, other reports found that gambling behaviors decreased on average; although, those with *prior* gambling problems reported an increase in their gambling [52,53]. In general, increases in web-based gambling were associated with COVID-19-related anxiety [54], feelings of isolation, and countering negative emotions (eg, being upset or restless) [55]. Many studies also reported increased COVID-19 risk in those with underlying SUDs [9,56,57], those with increased risky drug-seeking behaviors [58], and those with increased drug and alcohol use [59]. Our results suggest that those who gambled or sold drugs before or during the pandemic were at an increased risk for COVID-19, supporting the prior literature.

Per each subhypothesis, higher proportions of COVID-19 were observed with various other destructive behaviors. The subhypothesis *never vs. anytime* tested whether any history of destructive behaviors (between 1 month and >1 year ago) resulted in a higher proportion of positive COVID-19 tests or diagnoses at the time of the survey. The proportion of *test+* was higher with reports of 6 destructive behaviors: attention problems, bullying, gambling, violence, selling drugs, and destruction of property. The proportion of *diagnosis* was higher with reported histories of 17 of the 20 destructive behaviors. That is, those who reported any temporal history of these behaviors (between 1 month and >1 year ago) had higher proportions of positive COVID-19 tests or diagnoses. However, it is difficult to discern whether the behaviors themselves influenced SARS-CoV-2 infection or whether SARS-CoV-2 infection increased the preponderance for these behaviors. Future work should include the dates of SARS-CoV-2 infection to aid interpretability, although the data would remain retrospective.

Subhypothesis *never vs. >1 year* tested whether those with a history of destructive behaviors before the pandemic (>1 year ago) had a higher proportion of positive COVID-19 tests or diagnoses than those who never experienced destructive behaviors. Those who reported prepandemic gambling and drug selling reported a higher proportion of positive COVID-19 tests (*test+*; Table 6), and those who reported prepandemic attention problems, bullying, gambling, isolation related to substance use, and destruction of property reported more COVID-19 diagnoses (*diagnosis*; Table 6). These findings suggest that the participants who exhibited these behaviors before the pandemic had higher reports of SARS-CoV-2 infection. Research on other infectious diseases (eg, sexually transmitted diseases) has implicated similar risky or destructive behaviors in infection risk, including antisocial behaviors [11,12], rebelliousness [10], deviant behavior, aggression, and alcohol and drug use [13,15].

Subhypothesis *anytime and never vs. >1 year* tested whether a history of destructive behaviors before the pandemic (>1 year ago) resulted in a higher proportion of positive COVID-19 tests or diagnoses than those who experienced behaviors during the pandemic or those who never experienced destructive behaviors. The results closely mirrored those from the subhypothesis *never vs. >1 year*, although fewer behaviors were observed overall (Table 7). These results emphasize the pervasiveness of gambling and drug selling behaviors across all 3 subhypotheses and suggest that major problems relating to gambling and illegal drug distribution may greatly impact a person’s risk of SARS-CoV-2 infection.

Results from these proportion tests were subsequently used to build MVLR models and test how well GAIN-SS behaviors and covariates modeled, or predicted, the incidence of COVID-19.

### Destructive Behaviors Predict COVID-19 Infection

MVLR with iterative downsampling was used to test the predictive accuracy of destructive behaviors in modeling SARS-CoV-2 infection (Table 8). Ample research suggests that MVLR can either outperform or produce similar results to those produced by other machine learning (ML) approaches [60,61]. Proportion test results were used to select the predictors (independent variables) for each model. For all models, accuracies, sensitivities, specificities, PPVs, NPVs, and AUROCs were higher when modeling *diagnosis* than when modeling *test+*, and the behaviors included in *test+* models were always a subset of those included in *diagnosis* models. The outperformance of *diagnosis* models could be because of (1) the fact that COVID-19 tests were not initially widespread at the start of the pandemic and (2) the frequency of false positive COVID-19 tests. The participants who falsely tested positive for SARS-CoV-2 but did not exhibit GAIN-SS behaviors may have been included in the analyses, thereby skewing the data. Other scenarios are also possible where those who reported, or did not report, GAIN-SS behaviors may have (1) not been tested for SARS-CoV-2 but *were* infected or (2) been tested but received a false negative result. Given these considerations, a clinical diagnosis by a physician may have been more accurate to represent the true incidence of SARS-CoV-2 infection in this cohort.

Overall, the highest accuracy for modeling *diagnosis* was 95.55% and resulted when “attention problems” was included as a predictor in the *never vs. >1 year* model (Table 8). Previous research has implicated attention-deficit/hyperactivity disorder in COVID-19 risk, particularly in women [9]. This has been linked to that fact that those struggling with attention-deficit/hyperactivity disorder may have poorer access to health care, be living in population-dense environments, or have comorbid conditions [62]. Our results support these findings and demonstrate that a history of attention problems, as identified with the GAIN-SS, is an important predictor of COVID-19 diagnosis. This result was not confounded by covariates, given that the highest accuracy was 77.9% when only age, income, and education level were included in the model (Table S4 in Multimedia Appendix 1).

Gambling (accuracy=83.1%) and bullying (accuracy=83.7%) were also important predictors of *diagnosis* in the *never vs. >1 year* model. Problematic gambling and its relation to COVID-19 was discussed in the previous section. Although there is ample evidence of increased bullying during the COVID-19 pandemic [63,64], there is a lack of research identifying bullying behaviors as a potential COVID-19 risk factor. Given its importance in predicting COVID-19 *diagnosis*, we posit that persons who demonstrate this destructive behavior are more willing to risk the repercussions of their actions, which could translate to participating in activities that increase their risk for infection.

Accuracy was also high (95.1%) when 17 of the 20 destructive behaviors were included in the *never vs. anytime* model, suggesting that the demonstration of a wide variety of destructive behaviors both before and during the pandemic may be important COVID-19 risk factors. However, this data set lacked calendar dates for the reported positive COVID-19 tests (*test+)* and diagnoses (*diagnosis*), making it difficult to ascertain whether these behaviors were risk factors, or consequences, of SARS-CoV-2 infection.

### Limitations

This sample consisted of 366 participants, which is small for a population sample, and the sample size of those who responded “yes” for COVID-19 was approximately 10% (34-36/366), consistent with population estimates of COVID-19 in the United States at the time of data collection. Future work needs to assess larger population samples. Our sample was predominantly White (245/366, 66.9%), which is close to the current population estimates. Future work with a larger sample could ensure adequate sampling of population diversity. Downsampling may also be regarded as a limitation, as it reduces the count of training samples falling under the majority class to balance the counts of target categories. By removing some of the collected data, valuable information may be lost. However, resampling the downsampled majority class 1000 times facilitates sampling across the entire distribution, which may prevent some loss of information. The average of these resampled majority classes can thus represent the larger distribution. Finally, MVLR results do not represent true prediction, which would require adequately sized training and test sets; however, ample research demonstrates cases where MVLR either outperforms or mirrors results from widely used ML approaches [60,61]. Future work should incorporate larger sample sizes and should implement alternative ML approaches. These caveats aside, it must be noted that this study used iterative resampling to overcome a major confound that is common in current ML papers, namely overfitting [65,66].

### Conclusions

Results from this preliminary study implicate destructive and risk-taking behaviors in contracting SARS-CoV-2, specifically when the participants exhibited such behaviors before the pandemic. In general, gambling and selling drugs were most consistently observed in these relationships, but when modeling COVID-19 *diagnosis*, attention problems were also observed. The relevance of destructive and risk-taking behaviors in infection prediction suggests the importance of mitigation-related public messaging (eg, announcements and posters) at drug treatment centers and organizations involved in gambling (eg, casinos and bars). Making behavioral interventions more broadly available for those with destructive-type behavioral issues might also be important in this regard. Future work is needed to assess how these risk-taking behaviors might relate risk-taking in the context of SARS-CoV-2 infection (attending large gatherings, not wearing a mask, etc).

## Appendix

Multimedia Appendix 1 Includes supplemental methods, figures, and tables.

Multimedia Appendix 2 Unscored, uncoded data set with key.

## Abbreviations

AUROC: area under the receiver operating characteristic curve
GAIN-SS: Global Appraisal of Individual Needs–Short Screener
MH: mental health
ML: machine learning
MVLR: multivariable logistic regression
NPV: negative predictive value
PPV: positive predictive value
SUD: substance use disorder

## Data Availability

The unscored, uncoded data set analyzed for this study is available as Multimedia Appendix 2.
