# Supplementary material for: The Relationship Between a History of High-risk and Destructive Behaviors and COVID-19 Infection: Preliminary Study
Source: JMIR Form Res. 2023 Apr 14;7:e40821. doi: 10.2196/40821 (PMC10148215; doi:10.2196/40821)
Supplement: Multimedia Appendix 1 [file formative_v7i1e40821_app1.docx]

This is a Multimedia Appendix to a full manuscript published in the J Med Internet Res. For full copyright and citation information see http://dx.doi.org/10.2196/jmir.40821.

**Supplemental Methods**

*Methods: Proportion test hypotheses*

Proportion test hypotheses:

1. **GAIN-SS behaviors never exhibited vs. exhibited at any time = “*NEVER vs. ANYTIME*”**
   1. H_0_ (nondirectional): proportions did not differ between those responding “YES” and “NO” to test+ and diagnosis when GAIN-SS behaviors were coded as 0 = never and 1 = past month, 2-3 months, 4-12 months, 1+ years ago
   2. H_a_ (nondirectional): proportions differed between those responding “YES” and “NO” to test+ and diagnosis when GAIN-SS behaviors were coded as 0 = never and 1 = past month, 2-3 months, 4-12 months, 1+ years ago
   3. Ha (directional): the proportion of those responding “YES” to test+ and diagnosis was higher than those responding “NO” when GAIN-SS behaviors were coded as 0 = never and 1 = past month, 2-3 months, 4-12 months, 1+ years ago
2. **GAIN-SS behaviors never exhibited vs. exhibited more than one year ago (1+ years) = “*NEVER vs. 1+ YRS*”**
   1. H_0_ (nondirectional): proportions did not differ between those responding “YES” and “NO” to test+ and diagnosis when GAIN-SS behaviors were coded as 0 = never and 1 = 1+ years ago
   2. H_a_ (nondirectional): proportions differed between those responding “YES” and “NO” to test+ and diagnosis when GAIN-SS behaviors were coded as 0 = never and 1 = 1+ years ago
   3. H_a_ (directional): the proportion of those responding “YES” to test+ and diagnosis was higher than those responding “NO” when GAIN-SS behaviors were coded as 0 = never and 1 = 1+ years ago
3. **GAIN-SS behaviors more recently or never exhibited vs. exhibited more than one year ago = “*ANYTIME/NEVER vs. 1+ YRS*”**
   1. H_0_ (nondirectional): proportions did not differ between those responding “YES” and “NO” to test+ and diagnosis when GAIN-SS behaviors were coded as 0 = never, past month, 2-3 months, and 4-12 months and 1 = 1+ years ago
   2. H_a_ (nondirectional): proportions differed between those responding “YES” and “NO” to test+ and diagnosis when GAIN-SS behaviors were coded as 0 = never, past month, 2-3 months, and 4-12 months and 1 = 1+ years ago
   3. H_a_ (directional): the proportion of those responding “YES” to test+ and diagnosis was higher than those responding “NO” when GAIN-SS behaviors were coded as 0 = never, past month, 2-3 months, and 4-12 months and 1 = 1+ years ago

**Figure S1.** The 16 potential clinical diagnoses a participant could select. Participants indicating 10 or more of these diagnoses were excluded from analyses to maintain data integrity.


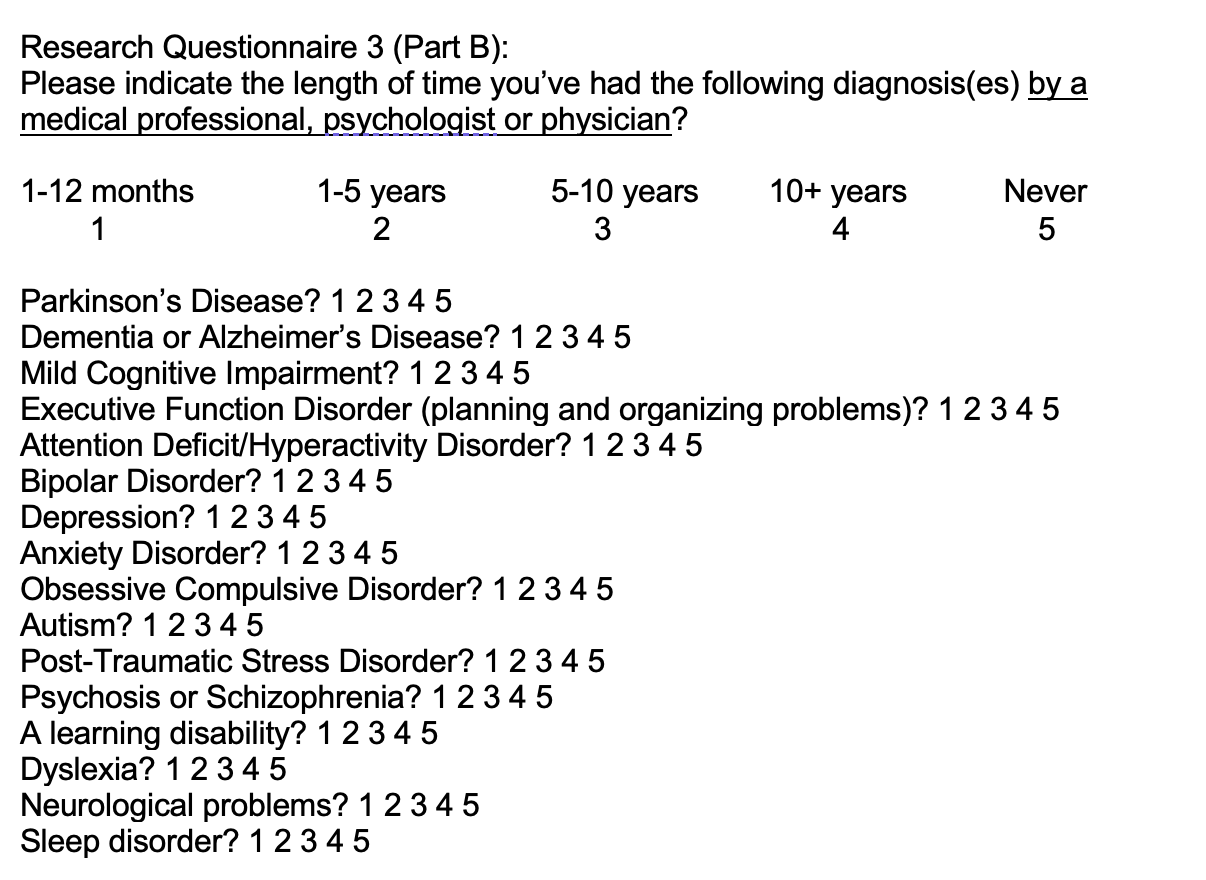


**Table S1**. Demographic summary. N indicates the sample size for each respective group; range is reported in years.

**Table S2.** Complete set of Wilcoxon Rank Sum tests results for demographic variables that vary by *test+* and *diagnosis*.

**Table S3.** Complete set of Wilcoxon Rank Sum tests results for GAIN-SSS scores and question responses that vary by *test+* and *diagnosis*.

**Figure S2**. Box plots of GAIN-SS score (**A-B**) and response (**C-D**) differences by COVID-19 *test+* and *diagnosis* (yes/no). Scores range from 0-8 and responses range from 0-4.

**Table S4**. MVLR results when only demographic variables were included in the model to predict *test+* and *diagnosis*.

**Figure S3**. Odds ratio and related metrics for each of the three sub-hypotheses. (A) “NEVER vs. ANYTIME”. (B) NEVER vs. 1+ YRS”. (C) NEVER/ANYTIME vs. 1+ YRS”. SE = standard error; $\alpha$= .05; OR = odds ratio; CI = 95% confidence interval.

A

B

C
